# Influence of monaural auditory stimulation combined with music on brain activity

**DOI:** 10.3389/fnhum.2023.1311602

**Published:** 2024-01-11

**Authors:** Ming Chang, Kenta Tanaka, Yasushi Naruse, Yasuhiko Imamura, Shinya Fujii

**Affiliations:** Vie, Inc., Kanagawa, Japan

**Keywords:** music, monaural auditory stimulation, brain activity, theta rhythm, mismatch negativity, totonou

## Abstract

**Introduction:**

Recently, the increasing attention to mental states and psychophysical health has fueled the research into methods that can aid in relaxation and recovery. Traditional methods like meditation and sauna, while effective, have their limitations; thus, the need for more accessible and convenient alternatives.

**Methods:**

Our innovative approach combines monaural beats with music, attempting to replicate the relaxing effects of a sauna in the auditory domain.

**Results:**

In comparison to normal music and silent condition, the power of the theta active band significantly increased when listening to our modified music. Furthermore, after listening to modified music, there was a significant increase in mismatch negativity (MMN) amplitude in the oddball task. Additionally, participants’ subjective responses to a questionnaire indicated significant changes in body relaxation and other metrics after listening to the processed music.

**Discussion:**

This state is considered similar to the “totonou” state, which manifests in physical and mental feelings of relaxation, pleasure, and mental clarity in the sauna. Thus, the present research proposes a convenient method for achieving relaxation, opening an avenue for individuals to customize their “totonou” music based on personal preferences.

## 1 Introduction

In recent years, the growing emphasis on mental wellbeing and holistic health has driven the search for methods that facilitate relaxation and refresh. Meditation, an age-old practice for relaxation and mind and body attunement, is increasingly recognized in modern society, as meditation not only helps individuals achieving profound states of relaxation but also amplifies the theta or/and alpha brain activity (Aftanas and Golocheikine, 2001; Takahashi et al., 2005; Cahn and Polich, 2006; Lagopoulos et al., 2009). However, its nuanced techniques and rigor can be challenging, leaving many struggling to genuinely immerse themselves in a meditative state.

Conversely, going to the sauna, an ancient practice for physical relaxation, is deeply ingrained in numerous cultures (Hannuksela and Ellahham, 2001; Kukkonen-Harjula and Kauppinen, 2006). Compared to meditation, saunas do not require any specific techniques and can be enjoyed by anyone. Interestingly, recent studies indicate that saunas not only facilitate physical relaxation but also significantly increase alpha brain activity (Cernych et al., 2018). Moreover, our recent research (Chang et al., 2023) found increased theta and alpha waves after three sauna sessions. Moreover, in the oddball task conducted before and after, there was an increase in mismatch negativity (MMN) amplitude and a reduction in P300 amplitude after the sauna. This emphasizes that saunas can promote relaxation and enhance perceptual awareness, improving the overall sense of wellbeing, referred to as “*totonou*” state in Japanese culture. The effect of meditation and sauna in theta and alpha activity reveals the complex interplay between relaxation, cognitive rest, and brain activity patterns.

In addition to meditation and sauna, music can help us relax and reduce stress (Pelletier, 2004; Koelsch and Stegemann, 2012; Thoma et al., 2013). The power of music, especially its impact on human emotions and cognition, has long been explored. Existing research has consistently demonstrated that music can’t only facilitate relaxation but also induce notable alterations in brain activity (Chanda and Levitin, 2013; Koelsch, 2014). However, there is limited empirical evidence on the specific enhancement of theta and alpha frequencies by musical stimuli.

Auditory beat stimulation (ABS) is a non-invasive neuromodulation method that presents a potential solution. It employs auditory stimuli to generate combination tones, encompassing binaural or monaural beats. These beats span across various frequency domains, such as theta (4–8 Hz), alpha (8–13 Hz), and gamma (30–50 Hz), etc. The primary objective of these auditory stimuli is to elicit a neural frequency following response in the listener (Pastor et al., 2002; Schwarz and Taylor, 2005; Becher et al., 2015). Monaural beats are generated by superimposing amplitude-modulated signals of closely related frequencies, which can be delivered to a single ear or both ears simultaneously. This phenomenon originates from the interference patterns created when two sound waves with slightly different frequencies are played within a singular auditory channel. The phenomenon is created when two tones with close frequencies are superimposed. This overlapping results in a new frequency, discerned as a rhythmic pulsation, representing the difference in frequency (Hz) between the two original tones (Oster, 1973). For example, combining tones of 400 and 405 Hz in a single channel results in a 5 Hz monaural beat. A previous study suggested that monaural beat stimulation may help regulating state anxiety and enhancing wellbeing (Chaieb et al., 2017). However, this approach has its limitations, as such auditory stimulation can be overly monotonous, potentially leading to discomfort or distraction.

Consequently, integrating “normal” music with monaural auditory stimulation might be a promising strategy to brainwave entrainment. This synergy might enhance the overall experience, making it more pleasant and potentially more efficacious in modulating specific brain active patterns, such as the alpha and theta frequency. Thus, this study aimed to devise a novel form of auditory stimulus by combining music with monaural beats, allowing individuals to reach a state of mental and physical wellbeing similar to going to the sauna. First, we composed a soothing piece of music. Next, after analyzing its frequency spectrum, we incorporated specific pure tones to enhance the brain’s alpha and theta wave activities. Subsequently, we compared the effects of our modified music, regular music, and a silent condition to validate the efficacy of our monaural beat-infused music and determine if it could replicate the benefits derived from a sauna session.

## 2 Materials and methods

### 2.1 Participants

Eight healthy participants (4 men, age 21–41 years old) were recruited for participation in the experiment. All participants were right-handed and none had hearing or speech disorders. The experiment consisted of three conditions: modified music (regular music combined with a specific pure tone, henceforth referred to as “music+” condition), regular music (hereafter referred to as “music” condition), and silence. Each participant received the three type of stimuli on different days. To ensure fairness and avoid bias, the order of participation in each condition was balanced among participants.

Informed consent was obtained from all participants prior to the study. All experimental procedures were approved by the Shiba Palace Clinic Ethics Review Committee. Additionally, all procedures were in accordance with the Helsinki Declaration of 1964 and its later amendments.

### 2.2 Procedure

**FIGURE 1:**
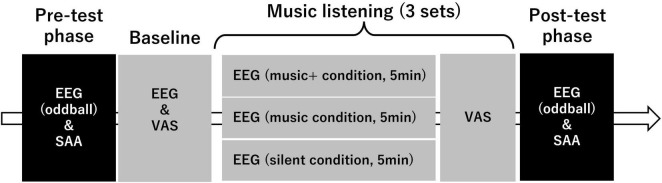
Flowchart of the experimental design and procedure. The figure outlines the sequence of the phases in the experimental protocol. The pre-test phase begins with electroencephalograms (EEG) assessment under an oddball paradigm followed by a salivary alpha-amylase activity (SAA). The baseline phase includes a similar EEG and visual analog scale (VAS) evaluation. The core of the experiment is the music listening phase. This phase is divided into three sets each including three conditions: modified music (“music+” condition) for 5 min, regular music (“music” condition) for 5 min, and silence (“silent” condition) for 5 min. VAS assessment is performed after each set. The post-test phase mirrors the pre-test with an EEG oddball assessment and SAA, which concludes the experiment. The arrow indicates the flow and sequence of the experimental components.

The experimental process included a pre-test phase, a music listening phase (or nothing in the silent condition), and a post-test phase (Figure 1). Both in the pre-test and post-test phase, we measured scalp electroencephalograms (EEG) and heart rate (HR) during an auditory oddball task. Additionally, we measured salivary alpha-amylase activity (SAA) to assess stress levels using a biosensor (Salivary Amylase Monitor, NIPRO Corporation, Japan) before and after music listening. Further, after the pre-test phase, we recorded 5 min of EEG data under silent conditions as baseline and conducted a questionnaire survey related to physical and mental states after the recording of EEG date.

The music listening phase was divided into three parts, each consisting of 5 min of music listening/silence followed by a questionnaire survey. The questionnaire used in this study was based on a visual analog scale (VAS) and implemented using MATLAB on a PC. It comprised 13 items (Table 1) extracted from our previous study. Items 1–10 were extracted from the “Altered States of Consciousness Rating Scale” (Studerus et al., 2010); for these items, a response of “No, not more than usually” was scored as 0, whereas a response of “Yes, much more than usually” was scored as 100. Items 11–13 were derived from a short-form self-report measure (S-MARE) (Sakakibara et al., 2014) to assess relaxation effects; for these items, a response of “No, I don’t think so” was scored as 0, while a response of “Yes, I think so absolutely” was scored as 100. Participants answered each question by moving the cursor along the score line (ranging 0–100 points) with a mouse and then clicking the left button to indicate their response. These items were chosen because they exhibited significant differences between before and after sauna bathing (Chang et al., 2023).

**TABLE 1:** Results of two-way repeated measures ANOVA for all questions in our questionnaires.

Questionnaires			Interaction (group × set)	Group effects	Set effects
**No, not more**		**Yes, much more**			
**than usually**		**than usually**	***F*-value (6,42)**	***F*-value (2,42)**	***F*-value (3,42)**
• Q1: I experienced boundless pleasure.			1.49	9.24**	6.53**
• Q2: Some everyday things acquired special meaning.			1.58	5.73**	3.72**
• Q3: I could see images from my memory or imagination with extreme clarity.			1.53	7.67**	3.28*
• Q4: I felt as if I was floating.			2.62*	9.06**	7.37**
• Q5: I saw brightness or flashes of light with closed eyes or in complete darkness.			0.93	6.84**	6.78**
• Q6: I felt isolated from everything and everyone.			3.57**	5.04*	1.76
• Q7: My muscles feel loose.			4.99**	18.86**	4.68*
• Q8: My breathing is faster than usual.			1.32	2.50	2.48 +
• Q9: I’m feeling very relaxed.			3.20*	9.27**	7.61**
• Q10: In the “totonou” state			1.38	3.04 +	2.42^+^
**No, I don’t**		**Yes, I think so**			
**think so**		**absolutely**			
• Q11: My heart is beating faster than usual.			1.13	2.44	0.49
• Q12: I have tension in my back.			1.86	1.06	1.27
• Q13: There’s no better feeling.			1.91	4.67*	1.71

*p < 0.05,

**p < 0.01.

### 2.3 Electroencephalographic recording

In the auditory oddball task, a series of stimuli (total, 200), consisting of standard tones at 1000 Hz (80% of the total) and infrequent “target” tones at 2000 Hz (20% of the total), with a 1.5 s onset of each stimulus were presented. These auditory stimuli, which lasted for 50 ms (with a 5 ms rise/fall time), were delivered through headphones at a sound pressure level of 60 dB. The stimuli were presented pseudo-randomly, ensuring that ≥2 standard stimuli were presented before each “target” stimulus. Participants were asked to press a response button with their preferred hand when detecting the target stimuli. Reaction times (RTs) were measured in ms, and the average of correct responses was calculated. EEG was recorded using a Wireless Biosignal Amplifier System (Polymate Mini AP108; Miyuki Giken Co., Ltd., Tokyo, Japan) and solid gel electrodes (METS INC., Chiba, Japan). We recorded from Cz, T3, and T4 electrodes at a sampling rate of 500 Hz, in accordance with the international 10–20 system. Additionally, ground and reference electrodes were placed on the left and right mastoids, respectively. Electro-ocular (EOG) activity was assessed using an electrode placed at the upper-outer edge of the left eye to measure eye blinks and vertical eye movements. An electrocardiogram was obtained using an electrode placed on the left upper chest to measure the HR. Recorded data were filtered using 0.1 Hz low and 40 Hz high cutoff filters.

During the oddball task, the epoch for the standard or target stimulus began 100 ms before stimulus presentation (baseline) and continued for 600 ms after stimulus onset. Epochs in which the EEG or EOG signal > ± 50 μV due to vertical eye movements and muscle contraction artifacts were automatically rejected. Then, standard and target event-related potentials (ERPs) were obtained by averaging the epochs for each stimulus. The difference waveform was calculated by subtracting the standard ERP from the target ERP. The following two time windows were determined according to a previous study (Chang et al., 2023): MMN (100–250 ms) and P300 (240–400 ms). MMN as the negative peak in the difference waveform and P300 as the positive peak in the difference waveform were calculated using these time windows. The area under the curve of the peak in the difference waveform (μV × ms) was determined to calculate the areas of MMN and P300.

At baseline and during the music listening/silence period, participants were asked to sit with their eyes closed for 5 min for brain activity measurement. Brain activity data were divided into equally sized segments (10 s) and fast Fourier transformations (FFT) were conducted. Each participant’s data were averaged across epochs for Cz, T3, and T4 electrodes, respectively, and the gross absolute spectral power for the theta (4–8 Hz) and alpha (8–12 Hz) frequency bands was computed.

### 2.4 Musical stimulation

**FIGURE 2:**
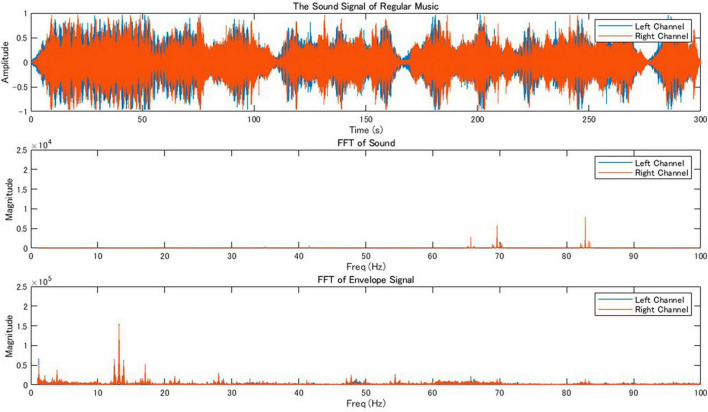
Audio signal analysis of regular music. The **top** panel displays the waveform of a stereo music track and shows the amplitude of the sound over time for both the left (blue) and right (orange) channels, covering a duration of 300 s. The middle panel presents the fast Fourier transformations (FFT) of the sound signal, revealing the frequency components present in the music for both channels in the range of 0–100 Hz, indicating the distribution of sound energy across the spectrum. The **bottom** panel shows the frequency response of the envelope, which represents slower changes in the amplitude of the sound, for both channels in the same frequency range.

One of the authors (KT) composed original music as stimulus for this study using Ableton Live software. The music featured bass, synthesizer, and piano sounds. We utilized sound instruments within the software: “Ambient Encounters Bass” for the bass, “Simple One Pad,” “The Greatest Pad,” and “Megaphatness Pad” for the synthesizers, and “Modular Pianos: 11 Tape Pianos” for the piano. The tones C (Do), C# (Do#), and E (Mi) were played using bass sounds to create low frequency growling sounds. The chord Cadd9, consisting of C (Do), E (Mi), G (Sol), D (Re) tones, was played using synthesizer sounds. The arpeggio melodies consisting of C (Do), D (Re), E (Mi), D (Re) were played with the piano sounds. The results of FFT and frequency analysis of the music envelope are shown in Figure 2. This original music served as stimulus for the music condition.

Here, we describe our novel auditory stimulus combining original music with pure tones. We define the peak frequency of the original music as θ_1_, and that of combining it with a pure tone as θ_2_; therefore, we can express the combined sound (*S*) as Eq. 1:


(1) $$S=\cos\theta_{1}+\cos\theta_{2}.$$

Then, we converted *S* to an analytical signal by the Hilbert transform and calculated its absolute values to obtain the envelope (*E*), expressed as


(2) $$E=\left|\cos\theta_{1}+\cos\theta_{2}+i\,\left(\cos\theta_{1}+\cos\theta_{2}\right)\right|.$$

Then, expanding the equation in Eq. 2 yields the following equation (Eq. 3), which indicates that the peak frequency of *E* is θ_1_−θ_2_.


(3) $$E=\sqrt{2+2\,\cos\left(\theta_{1}-\theta_{2}\right)}.$$

**FIGURE 3:**
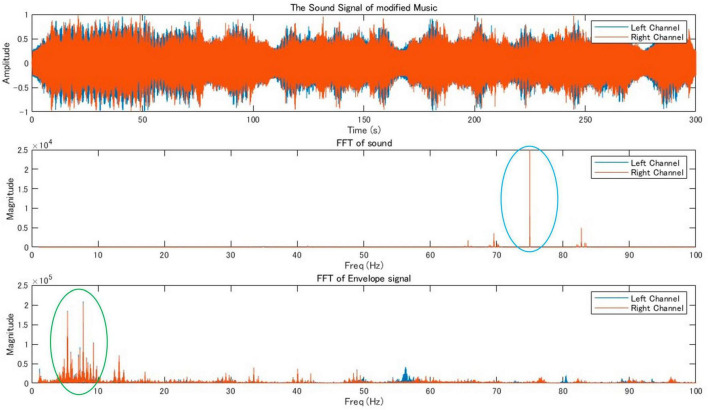
Audio signal analysis of modified music indicating the waveform and frequency analysis of a modified music track. The highlighted areas in the frequency domain (circled in blue and green) signify significant peaks that differ from the regular music signal by adding a 75 Hz pure tone, suggesting alterations in the frequency content or amplitude modulation characteristics in the modified music.

We found peaks around 82 Hz in the Fourier transform of the original music that we created (Figure 2). Therefore, θ_1_ of the original music was around 82 Hz. To shift the power peak of the music envelope into the theta band (around 7 Hz), we added a 75 Hz pure tone (θ_2_), to the original music. The results of FFT and frequency response of the envelope for the modified music is shown in Figure 3. This music was used as stimulus for the music+ condition. The music data are presented in Supplementary Data.

### 2.5 Statistical analysis

For the EEG data recorded during the oddball task in the pre-test and post-test phases, three-way analysis of variance (ANOVA) was conducted to determine the effects of the stage (pre-test vs. post-test) as a within-subject factor with two levels, conditions (music+ vs. music vs. silent) as a within-subject factor with three levels, and electrode location (Fz vs. T3 vs. T4) as a within-subject factor with three levels.

For the EEG data recorded during the baseline (pre) and music listening phases, a three-way ANOVA was conducted to determine the effects of the music listening set (pre vs. post 1 vs. post 2 vs. post 3) as a within-subject factor with four levels, conditions (music plus vs. music vs. silent) as a within-subject factor with three levels, and electrode location (Fz vs. T3 vs. T4) as a within-subject factor with three levels.

A two-way repeated measures ANOVA was conducted to evaluate changes in participants’ subjective feelings about their physical and mental states. The factors considered were conditions (music plus vs. music vs. silent) and music listening set (pre vs. post 1 vs. post 2 vs. post 3).

A two-way repeated measures ANOVA was conducted to evaluate other effects of music listening. The factors considered were conditions (music plus vs. music vs. silent) and stage (pre-test vs. post-test), which were expected to influence cognitive performance (measured by RT), SAA levels, and HR.

Here, as we aimed to examine the effects of listening to music, we only focused on the main effects of the set and their interactions. If significant effects were identified, the least significant difference method was employed as a *post-hoc* test for multiple comparisons within each repeated-measure ANOVA. The threshold for significance was set at *p* < 0.05.

## 3 Results

### 3.1 Effects of music on cognitive processing and neural oscillation

**FIGURE 4:**
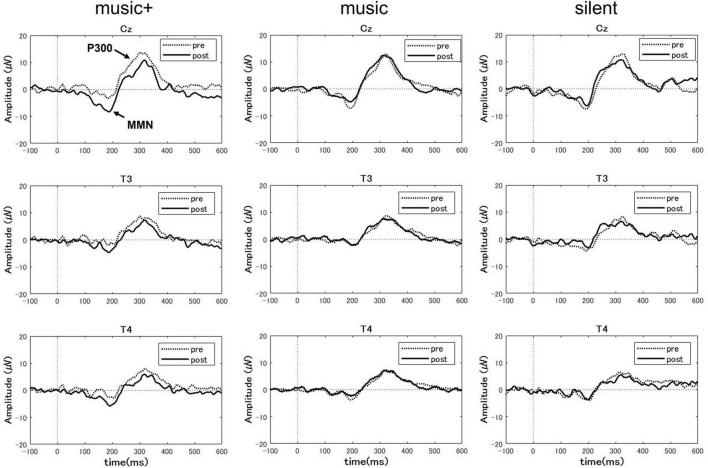
Grand-averaged event-related potential (ERP) difference waveform at three EEG sites (Cz, T3 and T4) for the auditory tasks before (pre) and after (post) music listening across the conditions. Each panel compares the pre-test (dotted line) and post-test phase (solid line) for each condition. The ERP components of P300 and mismatch negativity (MMN) are labeled at the Cz electrode site during the music+ condition. The waveforms exhibit the characteristic negative and positive peaks associated with auditory stimulus processing, with notable differences between the pre- and post-test phases.

We investigated potential changes in cognitive processing after music listening. Utilizing EEG data gathered during the pre-test and post-test phases, we compared the P300 and MMN amplitudes across participants under the three different conditions (Figure 4).

**FIGURE 5:**
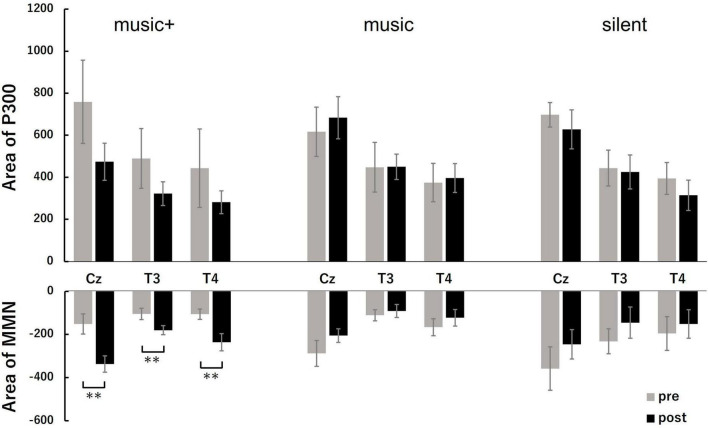
Quantitative analysis of MMN and P3 areas at three locations (Cz, T3, and T4) before (pre) and after music listening (post) across conditions. The **top** row shows the area of P300; the bars represent the mean values for the pre-test (gray) and post-test (black). The bottom row illustrates the area of MMN, following the same color coding for the pre- and post-test. The error bars indicate the standard error of the mean. **p < 0.01.

For the MMN area, the three-way ANOVA analysis revealed a significant main effect of electrode location [*F*_(2, 28)_ = 7.94, *p* < 0.01] and a significant interaction between condition and stage [*F*_(2, 28)_ = 10.23, *p* < 0.01]. No significant effects were observed for other interactions. The *post-hoc* tests indicated that, the MMN area significantly increased after listening to music [*F*_(1, 7)_ = 66.48, *p* < 0.01] only in the music+ condition. No significant differences were observed in the other conditions [music condition: *F*_(1, 7)_ = 1.82, n.s; silent condition: *F*_(1, 7)_ = 3.28, n.s; Figure 5]. Regarding the main effect of the electrode location, the results of multiple comparisons indicated that the MMN area at the Cz electrode location was significantly larger than that at the T3 and T4 electrode locations (MSe = 25315.0253, *p* < 0.05).

A three-way ANOVA with a within-subjects design conducted on P300 area data showed a significant difference only for the electrode location factor [*F*_(2, 28)_ = 25.77, *p* < 0.01]. No significant differences were detected for other factors or any interactions. Regarding the main effect of the electrode location, the results of multiple comparisons indicated that the P300 area at the Cz electrode location was significantly larger than that at the T3 and T4 electrode locations (MSe = 38928.3485, *p* < 0.05).

**FIGURE 6:**
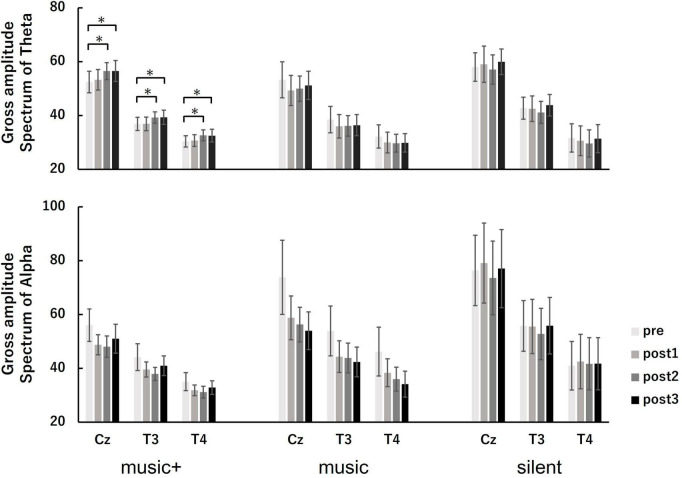
Amplitude spectral power of theta (**top** panel) and alpha (**bottom** panel) at baseline (pre) and each music listening set (post 1, post 2, and post 3) across the conditions. The measurements were taken at electrode sites Cz, T3, and T4. The error bars indicate the standard error of the mean. *p < 0.05.

We also evaluated changes in neural oscillations during music listening. Using EEG data gathered at baseline (pre) and throughout each music listening set, we computed the spectral power across the three conditions. Furthermore, we calculated and compared the overall absolute spectral power of the theta and alpha frequency bands for each condition (Figure 6).

Regarding the theta power, the three-way ANOVA revealed a significant main effect for electrode location [*F*_(2, 84)_ = 133.81, *p* < 0.01] and a marginally significant interaction between condition and set [*F*_(6, 84)_ = 1.95, *p* < 0.1]. No significant differences were found for other factors or any interactions. The analysis of condition × set interaction indicated a marginally significant in theta power on set was observed only in the music+ condition [*F*_(3, 21)_ = 2.65, *p* < 0.1]. However, no significance in theta power on set was observed in either the music [*F*_(3, 21)_ = 1.37, n.s] or silent conditions [*F*_(3, 21)_ = 0.68, n.s]. A simple main effects test for the set showed a significant increase of the theta power at post 2 and post 3 compared with pre (MSe = 22.3425, *p* < 0.05) in the music+ condition. Regarding the main effect of the electrode location, the multiple comparison results revealed that theta power was significantly greater at the Cz electrode location than at the T3 and T4 electrode locations and theta power was greater at the T3 electrode location than at the T4 electrode location (MSe = 104.4563, *p* < 0.05).

Regarding the alpha power, the three-way ANOVA revealed a significant interaction between set and electrode location [*F*_(6, 84)_ = 3.95, *p* < 0.01] and a marginally significant interaction between condition and electrode location [*F*_(4, 84)_ = 2.31, *p* < 0.1]. No significant differences were found for condition factors or the interaction between condition and set. The set × electrode location interaction analysis indicated a significant decrease in alpha power only at the Cz [*F*_(3, 21)_ = 3.83, *p* < 0.05] and T3 electrode locations [*F*_(3, 21)_ = 4.87, *p* < 0.01]. There was also a significant difference in the electrode location for all sets [pre: *F*_(2, 14)_ = 49.61, *p* < 0.01; post 1: *F*_(2, 14)_ = 44.99, *p* < 0.01; post 2: *F*_(2, 14)_ = 54.72, *p* < 0.01; post 3: *F*_(2, 14)_ = 52.02, *p* < 0.01]. A simple main-effects test for the set showed a significant decrease at post 1, post 2, and post 3 compared with the pre value at the Cz (MSe = 93.4595, *p* < 0.05) and T3 (MSe = 35.9184, *p* < 0.05) electrode locations. Furthermore, a simple main-effects test demonstrated that alpha power was significantly greater at the Cz electrode location than at the T3 and T4 electrode locations and theta power was greater at the T3 electrode location than at the T4 electrode location for all sets (pre: MSe = 96.8351, *p* < 0.05; post 1: MSe = 81.7024, *p* < 0.05; post 2: MSe = 60.9102, *p* < 0.05; post 3: MSe = 74.2329, *p* < 0.05).

### 3.2 Effects of music on cognitive performance and physiological parameters

**FIGURE 7:**
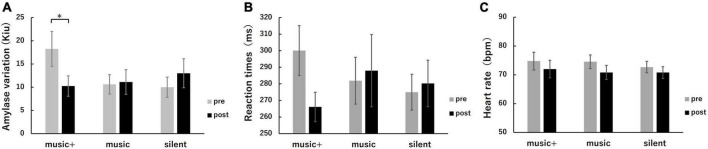
Physiological and cognitive response metrics across conditions. Average SAA **(A)**, RT **(B)**, and HR **(C)** were measured before (pre, gray) and after (post, black) music listening. The error bars indicate the standard error of the mean. *p < 0.05.

To evaluate potential changes in cognitive performance and physiological parameters following music listening, we calculated the average RT, SAA, and HR during the oddball task at both pre-test and post-test phases for all participants across each condition (Figure 7).

The two-way repeated measures ANOVA for SAA revealed a significant interaction between conditions and stages [*F*_(2, 14)_ = 4.32, *p* < 0.05]. No significant effects were observed for the group or any interactions. A simple main effects analysis for the stage indicated that the SAA significantly decreased only in the music+ condition [*F*_(1, 7)_ = 17.40, *p* < 0.01], while no significant change was observed in the other conditions [music condition: *F*_(1, 7)_ = 0.03, n.s; silent condition: *F*_(1, 7)_ = 0.82, n.s]. The two-way repeated measures ANOVA for HR showed no significant interaction between conditions and stages or any significant effects for any factor. The two-way repeated measures ANOVA for RT revealed no significant interaction between conditions and stages. Although a significant effect was observed for stages [*F*_(1, 7)_ = 10.79, *p* < 0.05], no significant differences were found among conditions [*F*_(2, 14)_ = 0.12, n.s].

### 3.3 Effects of music on subjective feelings

**FIGURE 8:**
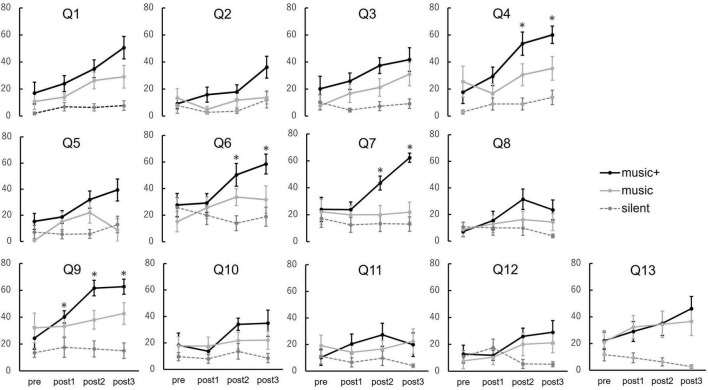
Subjective ratings for questionnaire items across conditions. The line graphs depict participant responses to the 13 items (Q1–Q13) evaluating subjective experiences. Average scores are computed for baseline (pre) and after each music listening set (post 1, post 2, and post 3). The conditions are represented as follows: music+ condition (black line), music condition (gray line), and silent condition (dotted line). The error bars indicate the standard error of the mean. *p < 0.05.

To evaluate the impact of music listening on participants’ subjective perceptions of their physical and mental states, we compared the questionnaire scores at baseline and after each music listening session for all participants across each condition (Figure 8).

The outcomes of the two-way repeated measures ANOVA are shown in Table 1. Out of all 13 questions, significant interactions were observed in the results of four questions. The analysis of condition × set interaction indicated that the average scores increased significantly only in the music+ condition. Specifically, these four questions are; “Q4: I felt as if I was floating” [*F*_(3,__21)_ = 6.70, *p* < 0.01], “Q6: I felt isolated from everything and everyone” [*F*_(3,__21)_ = 5.16, *p* < 0.01], “Q7: my muscles feel loose” [*F*_(3,__21)_ = 8.40, *p* < 0.01] and “Q9: I’m feeling very relaxed” [*F*_(3,__21)_ = 15.90, *p* < 0.01]. For the questions “My heart is beating faster than usual” and “I have tension in my back,” no significant interactions or main effects were observed. However, for the other questions, there was ≥1 significant main effect either in the set or condition factors.

## 4 Discussion

In this study, we developed an innovative auditory stimulation technique by merging music with monaural beats, aiming to emulate the therapeutic benefits of a sauna session. This unique blend, which superimposes specific pure tones onto a soothing music, is tailored to amplify the alpha and theta brainwave activities. By comparing the effects of our modified music, regular music, and silent conditions, we observed that only listening to the modified music significantly increased the MMN amplitude while the SAA levels significantly decreased. Concurrently, only participants exposed to this modified music demonstrated a significant enhancement in theta power. The questionnaire results further highlighted significant differences in the responses of participants under the monaural beat music condition compared to the other conditions.

In this study, the significant increase in MMN amplitude observed during the post-test phase aligns with previous findings from a sauna study (Chang et al., 2023). MMN is considered to be attention-dependent; its elicitation can occur regardless of whether participants consciously direct their attention toward the auditory stimulus (Näätänen et al., 1993; Alho et al., 1994; Tremblay et al., 1998). Thus, MMN is a reliable metric for pre-attentive auditory indices. The increase in MMN amplitude suggests an enhanced sensitivity to auditory stimuli. In addition, the results of the auditory oddball task indicated a slight reduction in both P300 amplitude and RT after listening to the modified music, although these changes were not as significant as those observed after sauna exposure. This result suggests that the impact of listening music might not be as pronounced as that of sauna sessions. Nevertheless, the lower number of participants for the music study compared to the sauna study could have influenced the statistical outcomes. We plan to increase the number of participants in future studies to enhance the generalizability of the findings. Furthermore, we observed that the MMN and P300 areas were larger at the Cz electrode than at the T3 and T4 electrodes. The absence of significant interaction between the stage and electrodes suggests that all three electrodes exhibited similar effects in response to listening to modified music and the difference in area between electrodes is unrelated to music listening. It has been previously reported that the neural generators of MMN include the auditory cortex in the temporal lobe (Picton et al., 2000) as well as the frontal lobe (Deouell, 2007), parietal lobe (Lavikainen et al., 1994), and subcortical region (Csépe, 1995). The intracerebral sources of P300 are present in various brain regions, particularly the temporal and parietal lobes (Polich, 2007). Thus, differences in the areas recorded at the Cz, T3, and T4 electrodes might be attributable to the varying distances between the electrodes and intracerebral sources of MMN and P300. Specifically, the Cz electrode is positioned closer to the intracerebral sources than the T3 and T4 electrodes.

In terms of neural oscillations, during music listening in the last two music sets, participants’ theta power significantly increased compared with that at the baseline; these findings are consistent with the findings of a previous sauna study (Chang et al., 2023). Regarding the questionnaire results for the statement “I feel very relaxed,” participants reported a stronger sensation during the music+ condition. This could be attributed to the enhanced theta power resulting from listening to modified music. The presence of theta waves suggests the onset of deep relaxation (Koudelková and Strmiska, 2018). Many advanced meditators exhibit increased theta wave activity during their practice, especially as they enter states of profound tranquility (Cahn and Polich, 2006; Lagopoulos et al., 2009). An increase in theta wave activity within the brain has been observed in various forms of meditation and relaxation practices (Jacobs and Lubar, 1989; Jacobs and Friedman, 2004). This is different from the alpha waves commonly associated with calm, relaxed wakefulness. When a person detaches from the external world and immerses in introspection, the brain is likely to enter a theta state. In the theta state, our sensory attention shifts from external stimuli to internal signals (Host’ovecký and Babušiak, 2017). The predominance of theta waves suggests that an individual is entering a state of profound relaxation and mental disengagement from the external environment, which is consistent with the results for the questionnaire item “I felt isolated from everything and everyone.” It has been suggested that when the brain is in a theta state, it can more easily connect distant ideas, facilitating creative thinking (Aftanas and Golocheikine, 2001). In addition, several studies have emphasized the crucial role of theta rhythm in cognitive processes (Klimesch, 1999; Slagter et al., 2009). Higher theta activity reflects increased awareness and attention as well as increased cognitive and emotional processing (Aftanas and Golocheikine, 2001). In this study, we enhanced participants’ theta power using modified music that targeted the theta frequency band. An increase in MMN was observed after participants listened to the modified music. Although the relationship between theta power and MMN has not been extensively explored, intracranial recordings during a flanker task suggest that oscillations in the theta range relate to variation in scalp ERPs (Cohen et al., 2008). Because MMN is a type of ERP associated with cognitive processes, these findings indicate that the observed increase in MMN amplitude during the post-test phase might be related to the enhanced theta activity during the music listening process.

In our experiments, only the music+ condition increased the power of the theta waves. However, unlike in the sauna research, alpha waves did not increase. This may be because the peak of the envelope of the modified music was around 7 Hz. Since 7 Hz falls within the theta wave band, the theta power should be enhanced, but the alpha power would not. On the other hand, the peak of the envelope for the music in the music condition is around 13 Hz, which would in principle increase alpha waves. However, according to our results, they did not increase but decreased. In contrast to the sauna study, the alpha power did not correlate with any experimental condition, showing a general decline across conditions. Since all experiments were conducted with participants’ eyes closed, participants may easily get drowsy. Some studies have indicated that as individuals transition from a wakeful state to a sleep state, alpha activity tends to decrease (Ogilvie, 2001; Silber et al., 2007). Thus, the reduction in alpha power might be attributed to participants gradually transitioning into sleep.

Regarding the questionnaire survey, we selected the questions from the sauna study for which significant interactions were observed. In other words, these questions reflect the subjective effects of the sauna. The present results indicated no significant changes only for the questions “My heart is beating faster than usual” and “I have tension in my back.” For other questions, there was ≥1 significant main effect either in the set or condition factors. This indicates that the sensation of an accelerated HR and tension in the back might be attributed to the alternating hot and cold effects experienced during the sauna sets. Our findings suggest that listening to music is unlikely to replicate these effects.

After completing the experiments under all conditions, we verbally asked the participants if they noticed any anomalies in the two pieces of music they heard. No one noticed any difference between the regular music and the modified music.

Despite the different trends in alpha activity changes between the music+ condition in the present study with the sauna condition in our previous study, there is consistency in the significant increase of theta activity and the significant increase in MMN amplitude. This suggests that, although not fully matching the “*totonou*” state, we may have successfully created a state somewhat close to it. This fusion strategy presents several advantages. First, it is an accessible relaxation method without the need for specialized settings such as saunas. Furthermore, integrating music with monaural beats did not only make the experience more enjoyable but in the future it could be modified to personal musical preferences.

Nevertheless, our study offers numerous directions for further development and exploration. While our study focused on replicating the benefits derived from a sauna session, future research could investigate its long-term effects and compare them with other relaxation methodologies. Moreover, a broader range of musical genres and styles could be applied to understand their varying impacts on brain activity. Last, to refine and optimize the technique for diverse populations, the individual variability in response to these auditory stimuli warrants further exploration.

In conclusion, our newly developed stimulation protocol for relaxation integrates the demonstrated benefits of music with ABS. As society gravitates toward holistic wellbeing, such innovations hold significant promise in enhancing mental health and overall wellbeing.

## Data availability statement

The original contributions presented in the study are included in the article/Supplementary material, further inquiries can be directed to the corresponding author.

## Ethics statement

The studies involving humans were approved by the Shiba Palace Clinic Ethics Review Committee. The studies were conducted in accordance with the local legislation and institutional requirements. The participants provided their written informed consent to participate in this study.

## Author contributions

MC: Writing – original draft, Data curation, Formal analysis, Investigation, Methodology, Resources, Software, Validation, Visualization, Writing – review and editing. KT: Resources, Software, Writing – review and editing. YN: Conceptualization, Methodology, Project administration, Supervision, Writing – review and editing. YI: Conceptualization, Funding acquisition, Methodology, Project administration, Supervision, Writing – review and editing. SF: Conceptualization, Investigation, Methodology, Project administration, Resources, Software, Supervision, Validation, Visualization, Writing – review and editing.
